# Probiotics may improve vaginal microbiota, metabolic disorders and ovarian function-related markers by modulating gut microbiota in POI mice

**DOI:** 10.1186/s12866-025-04097-y

**Published:** 2025-07-02

**Authors:** Jinxiang Cao, Wenjie Ma, Xiaoxia Chang, Danhua Pu, Rongrong Tan, Luanqian Hu, Tongtong Hong, Yugui Cui, Li Gao, Jie Wu

**Affiliations:** State Key Laboratory of Reproductive Medicine and Offspring Health, Department of Obstetrics and Gynecology, Jiangsu Province Hospital, The First Affiliated Hospital with Nanjing Medical University, Nanjing Medical University, Nanjing, Jiangsu China

**Keywords:** Premature ovarian insufficiency, Probiotics, Gut microbiota, Vaginal microbiota, Ovarian function, Metabolic disorder

## Abstract

**Background:**

Premature ovarian insufficiency (POI) is a condition identified by the decline in ovarian function before the age of 40. The treatment of POI patients deserves in-depth research. This study aimed to explore the impacts of probiotics on the gut and vaginal microbiota, ovarian function and metabolic indexes in a mouse model of POI.

**Methods:**

A mouse model of POI was successfully established by intraperitoneal injection of cyclophosphamide. Subsequently, the mice were divided into the control group, the POI group and the POI gavage group. Mice in the POI gavage group were gavaged for 28 consecutive days with a mixture of 12 probiotics. The anti-Müllerian hormone (AMH) and sex hormone levels, the number of follicles, the serum total cholesterol (TC) and triglycerides (TG) levels and the distributions of gut and vaginal microbiota of the mice were assessed and compared.

**Results:**

Compared with the healthy control group, the level of AMH and the number of growing follicles significantly decreased in POI mice (*P*<0.05), whereas the number of atretic follicles increased significantly (*P*<0.05). Meanwhile, the gut and vaginal microbiotas were disturbed in the POI group. Bacterial genera such as *Allobaculum*, *Prevotella* and *Bacteroides* were enriched in the gut microbiota, while *Proteus*, *Streptococcus* and *Rothia* were enriched in the vaginal microbiota. However, these bacteria contributed no favorable effect on the host. Interestingly, the increases in these taxa were reversed in POI mice treated with probiotics. Additionally, although there were no significant differences in AMH, estradiol (E2), and follicle stimulating hormone (FSH) levels between POI group without probiotics and POI gavage group (*P*>0.05), the AMH and E2 levels tended to increase while the FSH level tended to decrease in the gavage group. Besides, the number of growing follicles in the gavage group exhibited a slight increase compared with the POI group without probiotics (*P*>0.05), whereas the number of atretic follicles in the gavage group showed a decrease (*P*>0.05). Moreover, the levels of TC and TG in POI group without probiotics were elevated compared with the control group, while this trend was reversed in the POI gavage group (TG: *P*<0.05, TC: *P*>0.05).

**Conclusions:**

Probiotics may mitigate vaginal microbiota disruption, alleviate lipid metabolism disorders and improve indicators related to ovarian function by modulating the dysbiosis of gut microbiota in POI mice. These results provide some new directions and theoretical foundations for future microecological treatment in POI patients in clinical practice.

## Background

Premature ovarian insufficiency (POI) is a condition identified by the decline in ovarian function before the age of 40. The normal age of natural menopause for women is 40 to 50 years. POI can be considered premature menopause, but it is not a simple natural menopause; rather, it is a pathological process. The main manifestations of POI are abnormal menstruation (amenorrhea, sporadic menstruation or frequent menstruation), elevated gonadotropin levels ( FSH>25 U/L) and decreased estrogen levels [1].

Women with POI may experience hot flashes, night sweats, mood swings, vaginal dryness and other symptoms associated with menopause in the short term. They may also experience osteoporosis, cognitive impairment, an increased risk of cardiovascular disease and other problems in the long term. Therefore, POI can affect women’s health. In recent years, researches have documented a rise in the occurrence of POI. A study published in 2014 stated that the prevalence of POI among Chinese women is approximately 2.8% [2]. However, the incidence of POI has increased to about 3.7% according to recent international reports. POI places a heavy burden on patients’ body, mind and finances, and it is also a hot research topic in the field of gynecological reproductive endocrinology. A series of health problems caused by POI deserve our attention and warrant research, and an in-depth understanding of this disease will also contribute to societal harmony and stability.

Probiotics are a class of microorganisms that are beneficial to the host and are thought to have positive effects on gut health in both humans and animals. Probiotic mixture is a product made up of two or more probiotics and is often designed to provide more comprehensive microbial support. The benefits of probiotic mixture may vary depending on the specific combination of strains, but in general they are thought to have a range of beneficial impacts on the host’s health. Firstly, probiotic mixture can maintain intestinal microecological balance, which they do by promoting an increase in beneficial bacteria and limiting the growth of harmful bacteria [3]. This can be achieved through competition for ecological niches, production of antimicrobial substances, etc. Secondly, some strains in the probiotic mixture may be involved in the fermentation and decomposition of food, producing beneficial metabolites that promote digestion and absorption of food and improve nutrient utilization. Thirdly, probiotic mixture may enhance the functioning of the host immune system by activating host immune cells, such as macrophages and T-lymphocytes, as well as modulating the secretion of immune factors, thereby increasing resistance to infection and diseases [4]. Probiotics have also been found to be beneficial in restoring balance to the vaginal microbiota, which can help prevent vaginal infections and other complications [5]. Fourthly, some probiotics are thought to produce anti-inflammatory factors, such as short-chain fatty acids, as well as inhibit the release of inflammatory cytokines, which may provide some relief for conditions such as inflammatory bowel disease [6]. Fifthly, the probiotic mixture forms a protective barrier by adhering to the surface of the mucous membrane in the intestines, reducing the penetration of harmful substances and improving the defense function of the intestinal barrier so as to maintain intestinal health [7]. The sixth point is that some probiotics may regulate intestinal motility and function and maintain normal bowel movements by affecting the enteric nervous system, for example, by regulating the production and release of neurotransmitters and neuronal activity. The seventh point is that interactions between gut microbes and the brain may take place through the gut-brain axis, and the regulatory effects of probiotics may affect mood and mental health by influencing neurotransmitter production, inflammation levels, and other pathways [8, 9]. There are also probiotics that may have an effect on body weight and metabolism, helping to prevent obesity and related metabolic diseases [10, 11]. Last but not least, some probiotics may be helpful in maintaining cellular health by producing antioxidant substances, such as antioxidant enzymes and antioxidant small molecule compounds, which directly scavenge free radicals and reduce oxidative stress to achieve an antioxidant effect [12].

Previous investigations have shown that the gut microbiota and vaginal microbiota in individuals with POI differed from those in the healthy population, and the differences were associated with serum hormone levels [13–17]. These studies mainly focused on the correlation between POI patients and microecosystems and less on the treatment of POI. We speculate that the vaginal microbiota and systemic conditions might be improved by regulating the gut microbiota of individuals with POI to improve their quality of life. In the present study, we used POI model mice as research objects to investigate the effects of probiotics on the gut and vaginal microbiota, indicators related to ovarian function and lipid metabolism.

## Materials and methods

### Chemicals

The cyclophosphamide (CTX) used was produced by Meilunbio Company (powder, 1 g/bottle). During the experiment, 0.9% saline solution was injected at the indicated concentration.

### Probiotic preparation

The probiotic mixture used was produced by Zhenjiang Tianyi biotechnology co., LTD. The product (powder, 10 billion cfu/g, 100 g/bag) contained 12 kinds of probiotics (*Lactobacillus rhamnosus*,* Bifidobacterium lactis*,* Lactobacillus salivarius*,* Bifidobacterium longum*,* Lactobacillus coelicolor*,* Bifidobacterium youthfulum*,* Lactobacillus acidophilus*,* Lactobacillus paracasei*,* Lactobacillus reuteri*,* Streptococcus salivarius*,* Lactobacillus helveticus*,* Lactobacillus bulgaricus*) and of average ratio of AA. The origin of probiotic strains used in the experiment included those isolated from the human body as well as those isolated from traditional fermented foods, such as yogurt and kimchi. During the experiment, the indicated amount of this mixture was dissolved in normal saline and administered to the mice by gavage.

### Experimental animals and establishment of the POI mouse model

Specific-pathogen-free (SPF) level C57BL/6 mice (female, 6 weeks old, 18 ± 2 g, *n* = 28) were purchased from GemPharmatech. They were given unrestricted access to food and water continuously and were accommodated under suitable conditions of light (12 h light, 12 h dark), temperature (20–25 °C), and humidity (50 ± 5%). The mice were conditioned to grow to the age of 8 weeks. Twenty-eight female mice were allocated randomly into two groups: eleven in the healthy control group (HC) and seventeen in the model group (M). Approval for all animal experiments was granted by the Institutional Animal Care and Use Committee (IACUC) of Nanjing Medical University, with the assigned protocol number IACUC-2,208,025, and the experimental methods were performed according to the approved protocols. Mice were first anesthetized by intraperitoneal injection of 0.1 ml of 1% sodium barbiturate, blood was extracted from the medial canthal vein, followed by euthanasia by dislocating the head and neck of the mice unconsciously, and then finally tissue samples were taken.

Mice in the model group received a single intraperitoneal injection of 120 mg/kg CTX to construct the POI model [18], and mice in the healthy control group were concurrently injected with an equivalent volume of normal saline. After seven days of activity observation and weight recording, only one mouse in the model group died during the time, then blood of five mice in the model group and five mice in the healthy control group was collected after anesthesia for detection of serum AMH, and their ovarian tissues were collected for fixation to count follicle numbers.

### Probiotic gavage treatment

After verifying the success of POI mouse modeling, 11 mice remained in the model group and divided into 2 groups randomly: 5 mice in the POI group (P) and 6 mice in the POI gavage group (B). Mice in the POI gavage group were subjected to intragastric probiotic administration, the dose for which was calculated according to the equivalent dose for humans, i.e., 180 mg probiotic mixture per kilogram of mouse body weight, prepared as a solution in saline, 0.2 ml each day, for 28 consecutive days. Additionally, we classified the remaining 6 mice in the HC group as the control group (C). Then, the 6 mice in the control group and 5 mice in the POI group were given the same amount of normal saline for 28 days. The mice were weighed every three days, and the vaginal secretions and feces of each mouse were collected and stored at -80 °C after 28 days of intragastric probiotic administration for subsequent analysis.

### Measurement of serum AMH and sex hormone levels

After 7 days of modeling, blood was collected from 5 randomly selected mice in the healthy control group and model group after anesthesia. After 28 days of probiotic gavage, blood was taken from mice of the three groups (C, P, B) in the same way as described above. After allowing the samples to rest at room temperature for half an hour, serum was extracted by centrifugation at 4 °C and 2000 rpm, and the levels of AMH, E2 and FSH in the serum were measured by ELISA kits (Wuhan Cloud-Clone Corporation (Youersheng).

### Biochemical assays

After 28 days of intragastric probiotic treatment, mice in the POI group, the control group, and the POI gavage group were anesthetized, and then their blood was taken and left at room temperature for 30 min. The supernatant was extracted by centrifugation at 4 °C and 2000 rpm, and the serum levels of TG and TC were assessed using a total cholesterol assay kit and a triglyceride assay kit (Single reagent GPO-PAP method) from Nanjing Jiancheng Bioengineering Institute.

### Follicle counting

The ovaries of mice were extracted, preserved in formalin for 24 h, dehydrated through a series of ethanol concentrations, embedded in paraffin, and then sectioned into 4 μm thick slices, and took one out of every five slices. The sections were subjected to staining with H&E dye and examined using a light microscope. After staining, follicles were counted. An oocyte encircled by a single layer of flattened granulosa cells was categorized as a primordial follicle. An oocyte surrounded by a single layer of cuboidal granulosa cells was designated as a primary follicle. A secondary follicle was characterized by an oocyte encased in more than one layer of cuboidal granulosa cells without a discernible antrum. An antral follicle was identified as an oocyte enveloped by multiple layers of cuboidal granulosa cells and containing one or more antral spaces. An oocyte basically disintegration, granule nucleus shrinkage with disordered arrangement was classified as an atretic follicle. The findings were presented as the count of follicles per ovary.

### DNA isolation of feces and vaginal secretion and 16 S rRNA gene amplicon sequencing

Fresh fecal samples were gathered into sterile EP tubes immediately on defecation, avoiding contact with skin or urine of mice, and stored at -80 °C for subsequent analysis. A sterile cotton swab was slowly inserted into the vagina of the mice to obtain a full sample, and the head of the swab was cut off with sterile scissors and placed in a sterile EP tube, then stored at -80 °C for later analysis. Genomic DNA samples were extracted utilizing the OMEGA Soil DNA Kit (M5635-02) (Omega Bio-Tek, Norcross, GA, USA) according to the manufacturer’s guidelines and stored at -20 °C for subsequent analysis. The quantity and quality of extracted DNAs were assessed using a NanoDrop NC2000 spectrophotometer (Thermo Fisher Scientific, Waltham, MA, USA) and agarose gel electrophoresis, respectively. PCR amplification of the V3-V4 region of bacterial 16 S rRNA genes was conducted utilizing the forward primer 338 F (5’-ACTCCTACGGGAGGCAGCA-3’) and the reverse primer 806R (5’-GGACTACHVGGGTWTCTAAT-3’). PCR amplicons were purified using Vazyme VAHTSTM DNA Clean Beads (Vazyme, Nanjing, China) and quantified with the Quant-iT PicoGreen dsDNA Assay Kit (Invitrogen, Carlsbad, CA, USA). Following individual quantification, amplicons were evenly pooled, and pair-end 2 × 250 bp sequencing was carried out using the Illlumina NovaSeq platform with NovaSeq 6000 SP Reagent Kit (500 cycles) at Shanghai Personal Biotechnology Co., Ltd (Shanghai, China). The raw data for 16 S rRNA sequence was submitted to NCBI and the accession number was PRJNA1033792.

### Statistical and sequence analysis

All values were presented as mean ± standard deviation (SD). Differences in experimental data between the control and samples were evaluated using either one- or two-way analysis of variance (ANOVA). The Kruskal-Wallis test was employed for one-way ANOVA on the results. Data processing, analyses, and graphical representations were carried out using GraphPad Prism software, version 9.1 (San Diego, CA, United States; RRID: SCR_004812). P value in each figure indicate the significance, *P*>0.05 means no statistically significant, *P*<0.05 and *P*<0.01 means statistically significant. 

Analysis of sequence data was primarily conducted using QIIME2 and R packages (v3.2.0). Alpha diversity indices at the ASV level, including the Chao1 richness estimator, Observed species, Shannon diversity index, Simpson index, Faith’s PD, Pielou’s evenness and Good’s coverage were computed using the ASV table in QIIME2and presented as box plots. Ranked abundance curves at the ASV level were generated to assess the richness and evenness of ASVs across samples. Beta diversity analysis was conducted to explore the structural variation of microbial communities among samples utilizing Bray-Curtis metrics (Bray and Curtis, 1957), Jaccard metrics (Jaccard, 1908) and UniFrac distance metrics (Lozupone and Knight 2005, Lozupone, Hamady et al. 2007) and represented through principal coordinate analysis (PCoA), nonmetric multidimensional scaling (NMDS) and unweighted pair-group method with arithmetic means (UPGMA) hierarchical clustering [19].

## Results

### General conditions of the female mice after CTX injection

#### General condition and weight changes of mice in the control group and model group after 7 days of CTX injection

**Fig. 1:**
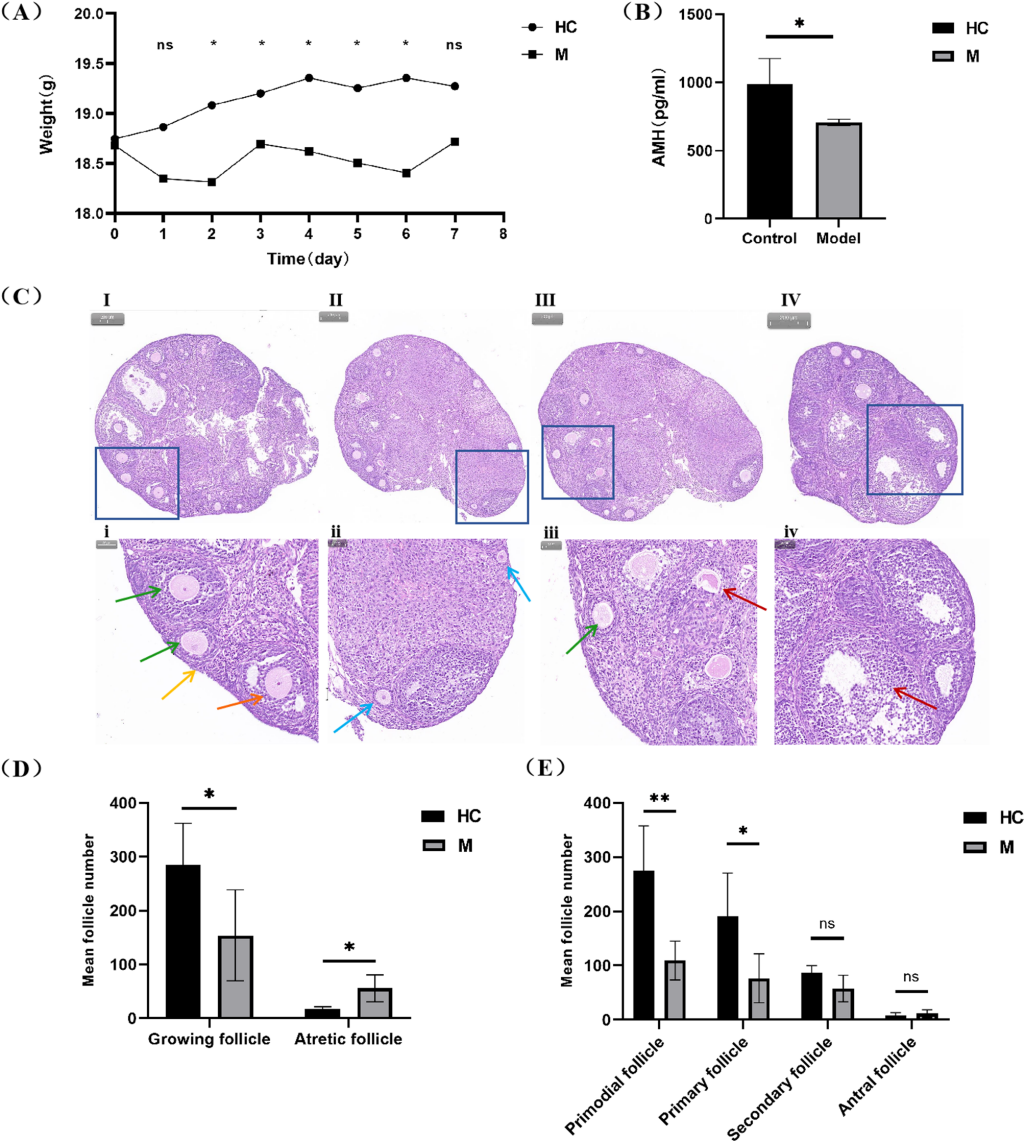
Weight changes of mice in the control group and the model group after 7 days of CTX injection (**A**). Levels of serum AMH in the healthy control and the model groups (**B**). H&E staining of follicles at various stages in mice subjected to CTX injection and normal saline. The primordial (yellow arrow), primary (blue arrow), secondary (green arrow), antral (orange arrow) and atretic (red arrow) follicles are annotated (**C**). (I and II, i and ii) show follicles of the healthy control group, (III and IV, iii and iv) show follicles of the model group. Scale bar = 200 µM in 4× and Scale bar = 50 µM in 20×. Where, the number of growing follicles and atretic follicles in the healthy control and model groups is (**D**) and the count of follicles at different stages in both the healthy control and model groups is (**E**). The results are represented as means ± SEM. ns means no statistically significant, a lone asterisk (*) denotes a significant difference at *P*<0.05, and double asterisks (**) indicate a significant difference at *P*<0.01

Most of mice in the model group survived well during and after administration, only one mouse died. From the first day after CTX injection, the mean body weight of mice in the model group started to decline, and it was notably lower compared to that of mice in the healthy control group from the 2nd day to the 6th day after CTX injection (*P*<0.05) (Fig. 1A).

#### Changes in serum AMH in the control group and model group after 7 days of CTX injection

The serum AMH level in the model group exhibited a significant reduction compared to that in the control group (*P*<0.05) (Fig. 1B).

#### Changes in follicle numbers in the control group and model group after 7 days of CTX injection

Follicle counting was conducted following H&E staining (Fig. 1C), and the categorization of follicles was carried out in accordance with methods described in a prior study [20]. The number of growing follicles in the model group was significantly lower than that in the control group (*P*<0.05), while the model group of mice showed a noteworthy elevation in the number of atretic follicles (*P*<0.05) (Fig. 1D). The quantity of primordial and primary follicles in the model group was notably less than that in the control group (primordial follicles: *P*<0.01, primary follicles: *P*<0.05); however, the secondary and antral follicles in the model group did not show a significant decrease when compared to those in the control group (*P*>0.05), but they did show a downward trend (Fig. 1E).

### General conditions of mice in the three groups after intragastric administration of probiotics

#### General condition and weight changes of mice in the control group, the POI model group and the POI gavage group

**Fig. 2:**
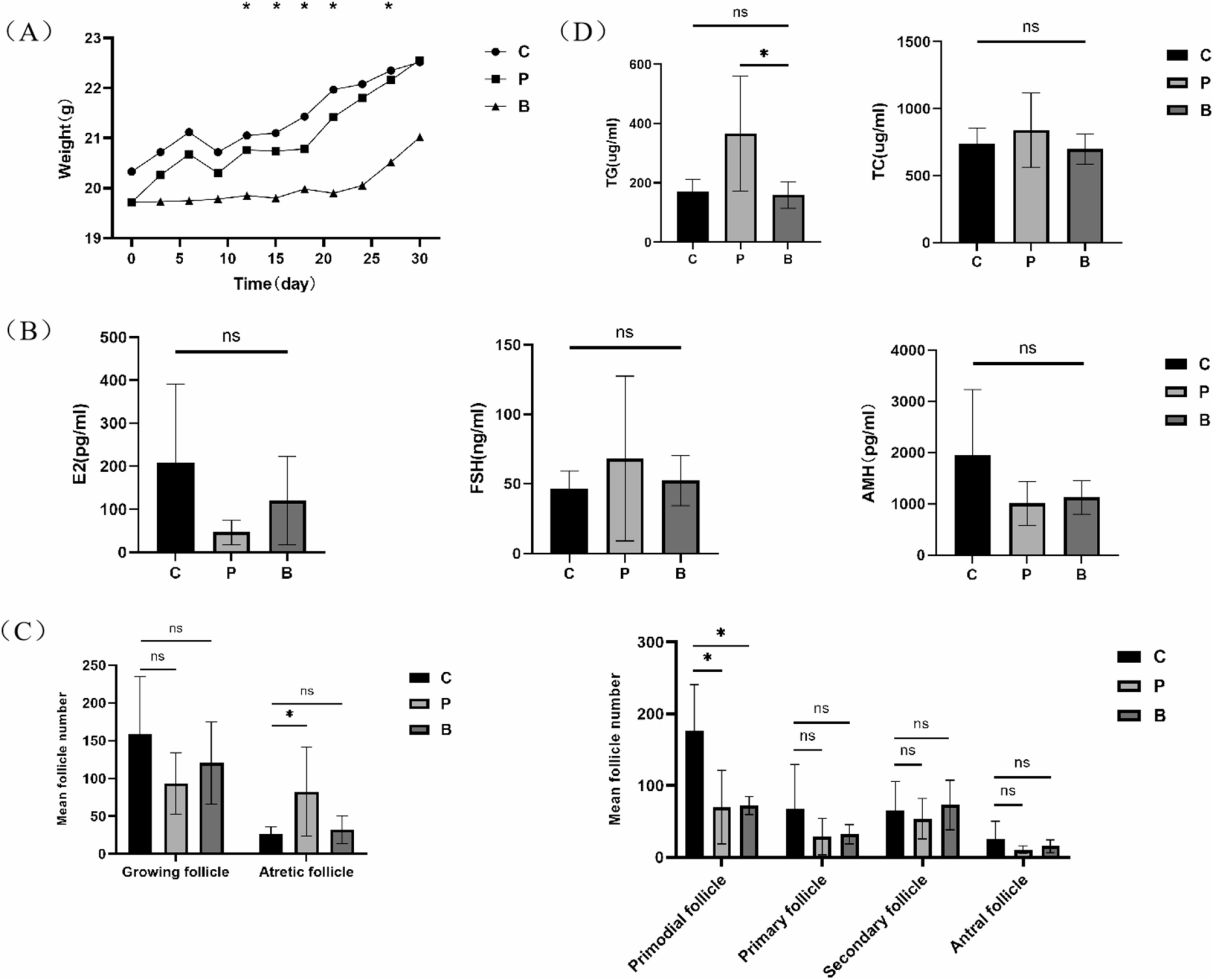
Weight changes of mice in the control group, the POI group and the POI gavage group over 28 days with gavage of probiotics (**A**). The levels of serum AMH in the control group, the POI group and the POI gavage group. The levels of serum E_2_ and FSH in the control group, the POI group and the POI gavage group (**B**). The number of growing follicles and atretic follicles in the control, the POI and the POI gavage groups. The number of follicles in each stage in the three groups (**C**). The levels of serum TG and TC in the control group, the POI group and the POI gavage group (**D**). The results are represented as means ± SEM. ns means no statistically significant, a lone asterisk (*) denotes a significant difference at *P*<0.05. **C**, the control group; P, the POI group; **B**, the POI with probiotic gavage group

Mice in the gavage group survived well during probiotic gavage, and no death was observed. The reaction and activity of mice in each group were normal, and no significant difference was observed. The body weight of mice in all groups increased, though the rate of increase was faster in the control group and the POI group (Fig. 2A).

#### Changes in serum AMH and sex hormones in each group

The serum E2 levels in both the POI group and the POI gavage group exhibited reductions in comparison to those in the control group (*P*>0.05), however, the E2 level in the gavage group was elevated in comparison to that in the POI group. The pairwise differences among the three groups were not statistically significant (*P*>0.05). Compared with that in the control group, serum FSH levels of mice in the POI model group and the POI gavage group were increased, which was more obvious in the former group. The difference between the control and gavage groups was not statistically significant (*P*>0.05). AMH levels were both decreased in both the POI group and the gavage group compared with that in the control group (*P*>0.05), but the AMH level in the gavage group was marginally higher than that in the POI group. The pairwise differences among the three groups did not reach statistical significance (*P*>0.05) (Fig. 2B).

#### Changes in follicle number in each group

The numbers of growing follicles in both the POI and POI gavage groups were not significantly smaller than those in the control group (*P*>0.05). However, mice in the gavage group exhibited slightly more growing follicles than those in the POI group. In contrast to the control group, mice in the POI group displayed a notable rise in the count of atretic follicles (*P* < 0.05); however, the number of atretic follicles in the gavage group did not show a statistically significant difference (*P*>0.05). These data showed that the number of growing follicles in the gavage group increased slightly compared with that in the POI group without probiotics, while the number of atretic follicles in the gavage group exhibited a significant decrease. These trends were similar to those in the control group (Fig. 2C).

The number of primordial follicles in mice from the POI group showed a statistically significant reduction of around 60% compared with that in the control group (*P*<0.05), while that in the POI gavage group was reduced by approximately 59%, which was also a significant decrease (*P*<0.05).

Compared with that in the control group, the number of primary follicles in mice of the POI group was reduced, but the difference was not statistically significant (*P*>0.05), and that in the POI gavage group was also reduced with no statistical significance (*P*>0.05).

In comparison to the control group, there was an approximately 17% reduction in the number of secondary follicles in mice from the POI group, while in the gavage group, the count was slightly higher than that in the control group. However, both differences were not statistically significant (*P*>0.05).

Compared with that in the control group, the numbers of antral follicles in mice in the POI group and the gavage group were reduced, but neither disparity reached statistical significance (*P*>0.05) (Fig. 2C).

#### Changes in serum total cholesterol and triglycerides in each group

The serum TG level in mice from the POI group exhibited a statistically significant increase compared to that in the control group (*P*<0.05). However, the serum TG levels of mice in the POI gavage and control groups were approximately equal, and the difference between them exhibited no statistical significance (*P*>0.05). In addition, the serum TC level of mice in the POI group was slightly higher than that in the control group, while the serum TC level of mice in the POI gavage group was slightly lower than that in the POI group. The distinction between the control and gavage groups did not reach statistical significance (*P*>0.05) (Fig. 2D).

### Comparison of the gut microbiota and vaginal microbiota in the three groups

#### Comparison of the diversity and composition of the gut microbiota in the control, POI and POI Gavage groups

(i) Alpha diversity

**Fig. 3:**
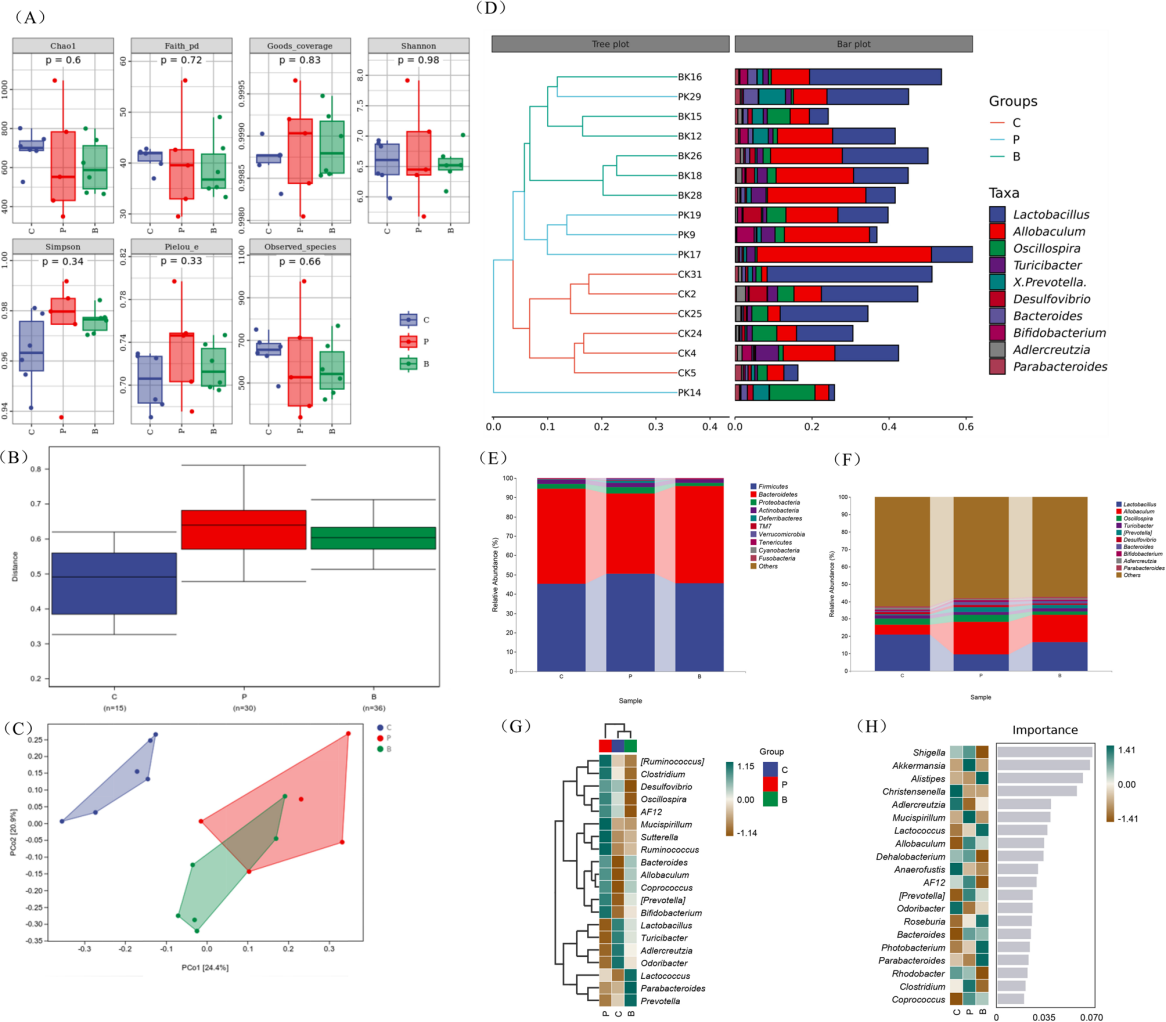
Alpha diversity index analysis diagram. Alpha diversity comparisons of the gut microbiota within the POI, POI gavage and control groups (**A**). Beta diversity comparisons of the gut microbiota in the POI, POI gavage and control groups. Variations in the gut microbiota of the three groups (**B**, **C**). Hierarchical clustering analysis showed different gut microbiota compositions for each sample at the genus level (**D**). Differences in relative abundance at the phylum level (**E**). Differences in relative abundance at genus level (**F**). Heatmap of microbial relative abundance at the genus level in the control, POI and POI gavage groups (*n* = 3) (**G**). Random forest analysis of the representative genera of each group (H). Colors represent the values (high values in green, low in brown). **C**, the control group; P, the POI group; **B**, the POI with probiotic gavage group

There was no significant difference detected in the alpha diversity of gut microbiota among the mice with POI, the POI gavage and the control (Fig. 3A), which means no significant difference could be found in the alpha diversity of gut microbiota between mice in either of the two groups (*P*>0.05). Chao1 estimator, Observed species, Faith pd, Goods coverage, Pielou’s index, Simpson index, Shannon index (*n* = 3). Data are depicted using box and whisker plots. And there were no significant differences among the control, the POI and the POI gavage groups.

(ii) Beta diversity

Beta diversity showed a significant distinction between the POI and control groups, as well as between the POI gavage and control groups, as indicated by ANOSIM (analysis of similarities, C vs. P: *R* = 0.672, *P* = 0.002; P vs. B: *R* = 0.395, *P* = 0.016; C vs. B: *R* = 0.739, *P* = 0.001) (Fig. 3B) and the results were displayed in PCoA plots (Fig. 3C). PCoA based on Bray-Curtis distance. The confidence was 0.95. Additionally, gut samples were subjected to a hierarchical clustering analysis. Notably, *Lactobacillus* was the dominant bacterial genus in the control group at the genus level compared to the POI group. *Allobaculum* was more abundant in the POI group than in the control group. However, in the POI gavage group, *Lactobacillus* was more dominant than *Allobaculum* (Fig. 3D). As can be seen in Fig. 3F, *Lactobacillus* has the highest percentage of the top 10 bacteria in terms of the top 10 bacteria detected individually, with levels of up to 20.1% in the control group, 9.7% in the POI group while *Allobaculum* was present in the POI group at levels up to 18.6% and 5.8% in the control group.

(iii) Comparison of the gut microbiota composition of mice in the control group, the POI group and the POI gavage group

The dominant gut bacteria in the three groups were *Firmicutes* and *Bacteroidetes* at the phylum level and *Lactobacillus* and *Allobaculum* at the genus level (Fig. 3E and F). At the phylum level, *Firmicutes* was more abundant in the POI group than in the other two groups, while the dominant phylum in the control and the gavage groups was *Bacteroidetes*, and the composition of bacteria in the gavage group approached that in the control. At the genus level, in comparison to the control group, the relative abundance of *Adlercreutzia* in the POI group exhibited a significant decrease (*P*<0.05), and there was also a decrease in the relative abundance of *Lactobacillus*, while that of *Allobaculum* was increased, but neither of these differences was statistically significant (*P*>0.05). In addition, compared with the POI group, the relative abundance of *Lactobacillus* was increased and that of *Allobaculum* was decreased in the POI gavage group, approaching the levels in the control group, however, neither of these differences was statistical significance (*P*>0.05).

(iv) Differences in the gut microbiota among the control group, the POI group and the POI gavage group

To further compare the differences in species composition among samples, we used a heatmap to analyze the species composition of each sample and determine the distribution trend of species abundance. Obviously, at the genus level, the distributions in gut microbiota of these groups are inconsistent. In comparison to the control group, the POI group exhibited reduced abundances of *Lactobacillus*, *Turicibacter*, *Adlercreutzia* and *Odoribacter*, alongside an increase in the abundance of *Mucispirillum*, *Sutterella*, *Ruminococcus*, *Clostridium* and *Bifidobacterium*, Probiotic gavage was able to reverse these changes in microbial abundances. Among them, the abundances of *Mucispirillum*, *Sutterella* and *Ruminococcus* could be reinstated to the level observed in the control group level (Fig. 3G).

Random forest analysis revealed that the POI gavage group alleviated the gut microbiota disorders caused by POI, as suggested by the reduced abundance of *Shigella*, *Mucispirillum*, *Dehalobacterium*, *AF12* and *Clostridium* and the increased abundance of *Alistipes*, *Lactococcus*, *Roseburia*, *Photobacterium* and *Parabacteroides* (Fig. 3H).

#### Comparison of the diversity and composition of the vaginal microbiota in the control, POI and POI Gavage groups

(i) Alpha diversity.

**Fig. 4:**
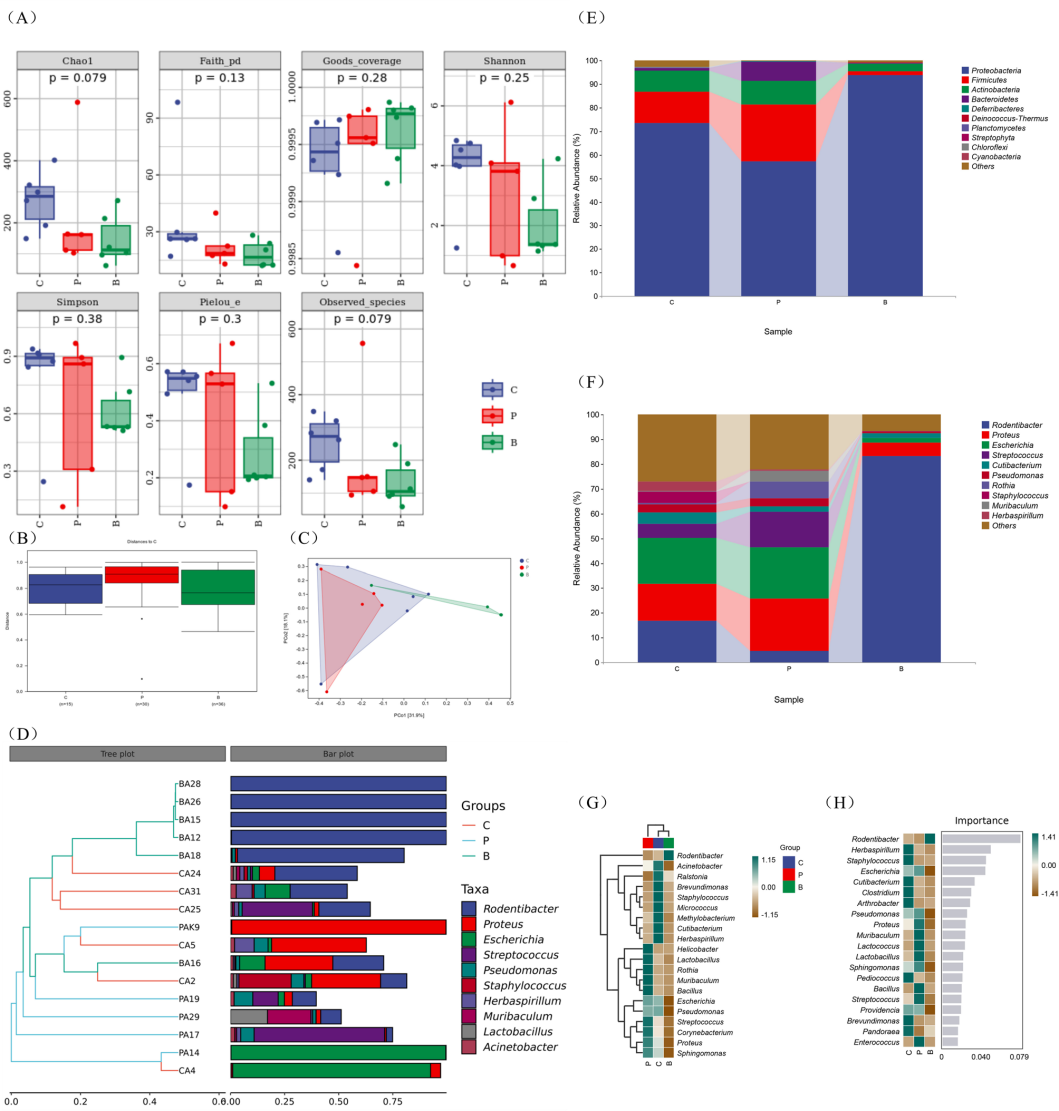
Alpha diversity index analysis diagram. Alpha diversity comparisons of the vaginal microbiota of the POI, POI gavage and control groups (**A**). Beta diversity comparisons of the vaginal microbiota of the POI, POI gavage and control groups. Variations in the vaginal microbiota of the three groups (**B**, **C**). Hierarchical clustering analysis showed different vaginal microbiota compositions of each sample at the genus level (**D**). Differences in relative abundance at the phylum level (**E**). Differences in relative abundance at the genus level (**F**). Heatmap of microbial relative abundance at the genus level in the control, POI and POI gavage groups (*n* = 3) (**G**). Random forest analysis of the representative genara of each group (H). Colors represent the values (high values in green, low in brown). **C**, the control group; P, the POI group; **B**, the POI with probiotic gavage group

There was no significant variance observed in the alpha diversity of the vaginal microbiota among the POI mice, the POI gavage mice and the control mice (Fig. 4A), which implies that there was no significant distinction in the alpha diversity of vaginal microbiota in mice of either two groups. (*P*>0.05).

Chao1 estimator, Observed species, Faith pd, Goods coverage, Pielou’s index, Simpson index, Shannon index (*n* = 3). Data are depicted using box and whisker plots. And there were no significant differences among the control, the POI and the POI gavage groups.

(ii) Beta diversity.

According to Anosim (analysis of similarities), there was no significant difference in beta diversity between the POI and control groups (*R* = -0.016, *P* = 0.503)., while that of the POI gavage and the control, that of the POI and the POI gavage group was significantly different (C vs. B: *R* = 0.326, *P* = 0.017; P vs. B: *R* = 0.493, *P* = 0.007) and the results were displayed in PCoA plots (Fig. 4B and C). PCoA based on Bray-Curtis distance. The ellipse confidence was 0.95. In addition, vaginal samples were subjected to a hierarchical clustering analysis. Notably, at the genus level, *Rodentibacter* was the dominant bacterium in the gavage group and most samples of the control group. *Rodentibacter* showed a pattern of dominance in mice vagina similar to that of *Lactobacillus crispatus* in the human vagina. The POI group showed higher abundances of *Proteus*, *Escherichia*, *Streptococcus* and *Muribaculum* (Fig. 4D).

(iii) Comparison of the vaginal microbiota composition of mice in the control group, the POI group and the POI gavage group.

At the phylum level, *Proteobacteria*, *Firmicutes*, *Actinobacteria* and *Bacteroidetes* were the predominant vaginal bacteria in all three groups., while *Rodentibacter*, *Proteus*, *Escherichia* and *Streptococcus* at the genus level (Fig. 4E and F). At the phylum level, *Firmicutes* was more abundant in the POI group than in the other two groups, while the dominant phylum in the control and the gavage groups was *Proteobacteria*, and the composition of bacteria in the gavage group approached to that in the control. At the genus level, in contrast to the control group, the POI group exhibited an increase in the relative abundance of *Proteus*, whereas the abundance of *Rodentibacter* was decreased (*P*>0.05). However, compared with the POI group, the relative abundance of *Rodentibacter* was significantly increased (*P*<0.01) in the POI gavage group, while the relative abundance of *Proteus* was decreased (*P*>0.05). This showed that probiotic administration via gavage was able to revert the alterations in these microbes. Among them, *Rodentibacter* could recover to higher level than that in the control group (Fig. 4G). Random forest analysis revealed that POI gavage alleviated the vaginal microbiota disorders caused by POI, as suggested by the reduced abundance of *Escherichia*, *Pseudomonas*, *Proteus*, *Muribaculum*, *Sphingomonas* and *Providencia* and the increased abundance of *Rodentibacter* (Fig. 4H).

#### Association between differentially abundant gut and vaginal microbes and clinical characteristics

**Fig. 5:**
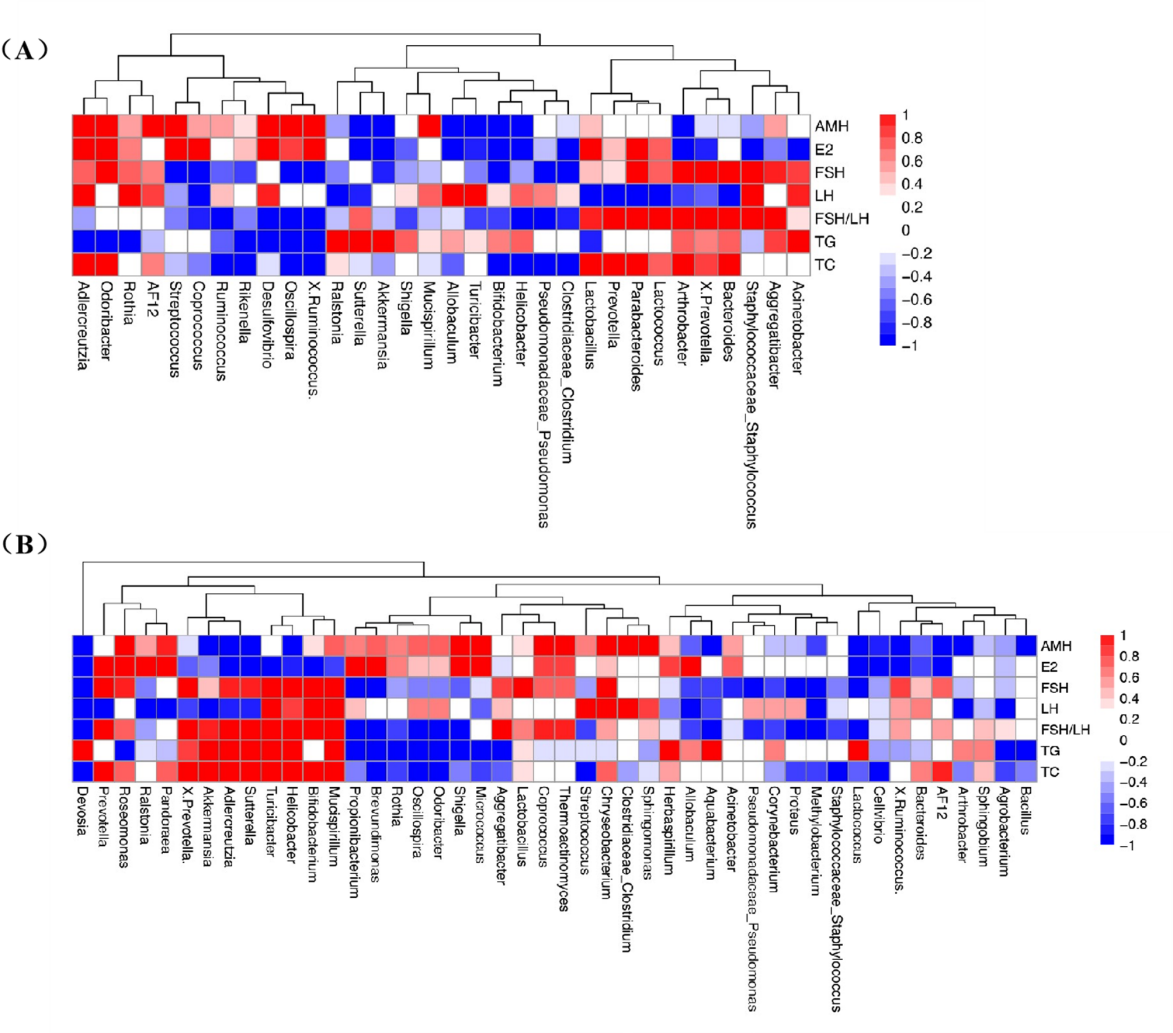
Associations between gut microbiota and serum indicators (**A**). Associations between vaginal microbiota and serum indicators (**B**)

Pearson correlation analysis was conducted to assess the connection between distinct gut microbes and serum hormones (Fig. 5A). The findings indicated a significant negative correlation between the AMH level and the relative abundance of *Turicibacter* (*R* = -0.53, *P* = 0.03). The E2 level was significantly positively correlated with the relative proportion of *Odoribacter* (*R* = 0.49, *P* = 0.04) and negatively correlated with the proportion of *Akkermansia* (*R* = -0.52, *P* = 0.03). Luteinizing hormone (LH) was significantly negatively correlated with *Parabacteroides* (*R* = -0.54, *P* = 0.03). The FSH/LH ratio exhibited a significant positive correlation with the relative abundance of *Aggregatibacter* (*R* = 0.55, *P* = 0.02), X.Prevotella. (*R* = 0.49, *P* = 0.046), *Bacteroides* (*R* = 0.63, *P* < 0.01) and *Parabacteroides* (*R* = 0.49, *P* = 0.047) but inversely associated with the proportion of *Oscillospira* (*R* = -0.49, *P* = 0.04). TG levels were significantly positively correlated with *Akkermansia* (*R* = 0.54, *P* = 0.02) and inversely associated with the relative proportions of *Adlercreutzia* (*R* = -0.51, *P* = 0.04) and *Odoribacter* (*R* = -0.51, *P* = 0.04). The TC level was significantly negatively correlated with the relative proportion of *Ruminococcus* (*R* = -0.54, *P* = 0.03).

Additionally, Pearson correlation analysis was also performed to assess the correlations between the differentially abundant vaginal microbes and serum hormones (Fig. 5B). The findings revealed a significant positive correlation between the AMH level and the relative proportions of *Micrococcus* (*R* = 0.52, *P* = 0.03) and *Roseomonas* (*R* = 0.50, *P* = 0.04). The E2 level exhibited a significant positive correlation with the relative proportions of *Pandoraea* (*R* = 0.65, *P* < 0.01) and *Roseomonas* (*R* = 0.49, *P* = 0.04). FSH was significantly positively correlated with *Turicibacter* (*R* = 0.50, *P* = 0.04) and *Bifidobacterium* (*R* = 0.52, *P* = 0.03) but negatively correlated with *Devosia* (*R* = -0.66, *P* < 0.01). LH was significantly positively correlated with *Bifidobacterium* (*R* = 0.50, *P* = 0.04) and *Chryseobacterium* (*R* = 0.52, *P* = 0.03). The FSH/LH ratio exhibited a significant negative correlation with the relative proportions of *Proteus* (*R* = -0.49, *P* = 0.04) and *Devosia* (*R* = -0.56, *P* = 0.02). The TC level showed a significant positive correlation with the relative proportions of *Turicibacter* (*R* = 0.50, *P* = 0.04), *Bifidobacterium* (*R* = 0.50, *P* = 0.04) and *Prevotella* (*R* = 0.53, *P* = 0.03).

## Discussion

The mouse model of POI excluded the confounding factors observed in human sample studies (including environmental exposure factors, sample collection and other human factors), and the POI model was constructed by applying the developed scheme reported in the literature [19]. Based on this model, we further explored the changes in serological indexes and gut and vaginal microecology of mice in the control group, the POI group and the POI gavage group with probiotics. Probiotic administration directly affected the gut microbiota of the mice. The findings revealed a significant alteration in the gut microbiota of mice in the POI group following probiotic administration, with a subsequent restoration to levels comparable to those of the control group. Compared with mice in the POI group without gavage, the indexes related to ovarian function, such as AMH and estradiol levels, were improved in the POI gavage group. The gavage group exhibited a slight increase in the number of growing follicles compared to the POI group without probiotics, whereas the number of atretic follicles in the gavage group showed a significant decrease. These trends were similar to those of the control group. Moreover, after administration of probiotics, the serum TG and TC levels of mice decreased slightly, indicating that metabolic disorders might be alleviated. In terms of the vaginal microbiota, the abundance of beneficial bacteria in mice from the gavage group showed a significant increase compared to that in the POI group, surpassing even the levels observed in the control group. These findings suggested that probiotic administration might alleviate ovarian dysfunction, metabolic disorders and imbalances in the vaginal microbiota of POI model mice to a certain extent by improving the gut microbiota.

In the context of reproductive endocrine diseases, research on the correlations with gut microbiota disorders is still in its infancy. Previous researches have indicated a correlation between disruptions in gut microbiota and alterations in the vaginal environment and are also involved in the occurrence, the progression and outcomes of diverse reproductive endocrine disorders, including conditions like polycystic ovary syndrome (PCOS) and endometriosis. According to previous reports, the mechanisms underlying gut microbiota disorders involved in reproductive endocrine diseases may involve the participation of the gut microbiota in host humoral and cellular immunity or gut disorders that affect the central neurotransmitters. The gut microbiota, both directly and indirectly, is involved in the regulation of sex hormones. Gut microecological stability plays a crucial role in the host nervous system, endocrine system and systemic diseases. Recently, numerous investigations have demonstrated the crucial role of the vaginal microbiota in preserving the health of the female reproductive system [21–23]. The gut and vaginal microbiota in individuals with POI exhibited significant alterations compared to those healthy individuals [24, 25]. Furthermore, probiotics were found to modulate the gut microbiota, offering potential control over various gastrointestinal conditions and fostering overall well-being. Probiotics confer metabolic advantages by enhancing the integrity of the gut barrier and mitigating inflammation, both of which are pivotal in the onset of age-related diseases [5, 26, 27]. These are similarities between the current study and the results in earlier studies.

In addition, there exists an intricate and intimate interrelationship between the gut microbiota and the vaginal microbiota, and this association involves multiple levels of immune regulation, circulation of microbial metabolites, and potential infection transmission. It has been found that the modulatory effects of gut microbes on the immune system may have distant effects by influencing the vaginal immune system [28], and Omenetti et al. noted that alterations in gut microbiota may have remote effects on vaginal immunity through the regulation of immune cells [29]. In terms of microbial metabolic circulation, metabolites generated by microorganisms in the intestines, such as short-chain fatty acids., may reach the vagina through the blood circulation and exert an influence on the vaginal microenvironment [30]. Another study found that there is a circulation of metabolites between the gut and the vagina, which may affect vaginal health through potential systemic regulatory mechanisms [31]. In addition, similarities between the gut and vaginal microbiota may be associated with the development and transmission of infectious diseases, but it has also been suggested that such similarities may have a role in protection from infection. Other studies have suggested that similarities between gut and vaginal microbiota may play a role in defensive immunity, but this is an area that still needs to be supported by more in-depth research [32].

However, to date, no studies have reported the effects of probiotics on improving ovarian function-related markers and alleviating vaginal microbiota disorders by regulating the gut microbiota. Therefore, our study on the alleviation of POI and its complications by regulating the gut microbiota is of great significance.

In terms of the experiments, based on the literature and considering that the method was similar to the clinical application of chemotherapy drugs in the population, only one intraperitoneal injection of CTX was selected to construct the POI mouse model, ensuring that the status of ovarian function was basically stable in the constructed model. Furthermore, the microbiotas of humans and mice are not identical, and the gut microbiota of healthy women consists mainly of *Firmicutes*, while the vaginal microbiota consists mainly of *Firmicutes*, especially *Lactobacillus*. In the mice in our study, the dominant gut microbes were *Firmicutes* and *Bacteroidetes* at the phylum level and *Lactobacillus* and *Allobaculum* at the genus level, while the most abundant genus in the vagina was *Rodentibacter*. Maintaining gut homeostasis relies on achieving a balance between *Bacteroidetes* and *Firmicutes* [33]. Estrogen stimulates hyperplasia and elevated glycogen production [34], and *lactobacilli*, the predominant bacteria in the vagina, can convert glycogen to lactic acid. This process contributes to maintaining the acidic environment of the vagina, suppressing the proliferation of pathogens, and fortifying the immune system [35, 36], which can be thought of as self-cleaning of the vagina. Notably, *Rodentibacter* showed a pattern of dominance in the mouse vagina similar to that of *Lactobacillus* in the human vagina.

By restoring the balance of the gut and vaginal microecosystem, probiotics may also improve the overall health and well-being of model mice with POI induced by chemotherapy. The results of animal experiments indicated that POI patients might modulate their gut microbiota by applying probiotics to improve vaginal microecology and metabolic indicators after the occurrence of ovarian dysfunction; this could help alleviate clinical symptoms such as atrophic vaginal inflammation and metabolic syndrome and improve their long-term quality of life. In addition, the effects on bone mass and cardiovascular disease can be further studied.

There are also some limitations to this study. Our results revealed the correlation between POI and the gut and vaginal microbiota in mice, and we found that probiotics could improve the microecology and related indicators of ovarian function and metabolism in mice, but this study lacked an in-depth exploration of specific mechanisms. According to the results of this study, the application of probiotics could alleviate lipid metabolism disorders, improve indicators related to ovarian function and mitigate vaginal microbiota disruption in POI model mice, providing a reference treatment strategy for the long-term improvement of the quality of life of patients with chemotherapy-induced ovarian insufficiency. In addition, our study also laid a foundation for other etiological studies of POI, including studies of genetic factors, immune factors, idiopathic factors and other iatrogenic factors. In the future, we can explore the relationship between POI caused by different etiologies and microecology. Furthermore, our findings establish the groundwork for uncovering the interaction between gut and vaginal microbiota in relation to POI. In the future, the composition of gut and vaginal microecology and the metabolome of POI patients can be studied through metagenomic sequencing through the multicenter collection of large samples of human specimens, and representative differentially abundant metabolites can be identified to guide diagnosis. Additionally, the vaginal or gut microorganisms of POI patients can be transplanted into sterile mice to investigate the potential causal relationship between POI and the microbiota and to study the underlying molecular mechanism.

## Conclusions

In this study, we first established a mouse model of POI and administered probiotics to mice with POI. The findings indicated that, after the application of probiotics, the gut and vaginal microbiotas of mice in the POI gavage group changed significantly and became similar to those of the control group. At the same time, the related indicators of ovarian function and metabolism in the POI gavage group also improved. Based on the results of our study and clinical observations, mice with chemotherapy-induced POI might benefit from taking oral probiotics to modulate their gut microbiota, which could mitigate the vaginal microbiota disruptions, alleviate lipid metabolism disorders, and improve indicators related to ovarian function.

Our results illustrate the therapeutic potential of probiotics in treating POI, providing new insights into the correlation between the gut microbiota and overall health, the pathogenesis and treatment of POI, the mechanisms underlying novel therapeutic strategies, and the application of probiotic-based therapies. Further clinical exploration of this strategy is warranted and we are currently conducting clinical studies in humans. In the future, in-depth mechanistic exploration can be continued to investigate the molecular mechanisms underlying the interaction between microecology and POI, laying a foundation for more extensive research on microecology and female reproductive endocrine diseases.

## Data Availability

The raw data for 16S rRNA sequence in this study are available in NCBI, the accession number was PRJNA1033792.

## Abbreviations

AMH: Anti-Müllerian hormone
CTX: Cyclophosphamide
E2: Estradiol
FSH: Follicle stimulating hormone
IACUC: Institutional Animal Care and Use Committee
LH: Luteinizing hormone
PCOS: Polycystic ovary syndrome
POI: Premature ovarian insufficiency
SPF: Specific-pathogen-free
TC: Total cholesterol
TG: Triglycerides
